# Effect of Microglial Activity on Gut Microbiota in Rats with Neuropathic Pain

**DOI:** 10.3390/ijms26115181

**Published:** 2025-05-28

**Authors:** Seung-Wan Hong, Liyun Piao, Eun-Hwa Cho, Eun-Hye Seo, Seong-Hyop Kim

**Affiliations:** 1 Department of Anesthesiology and Pain Medicine, Konkuk University Medical Center, Konkuk University School of Medicine, Seoul 05030, Republic of Korea; 2 Department of Infection and Immunology, Konkuk University School of Medicine, Seoul 05029, Republic of Korea; 3 Korea mRNA Vaccine Initiative, Gachon University School of Medicine, Incheon 21565, Republic of Korea; 4 Department of Medicine, Institute of Biomedical Science and Technology, Konkuk University School of Medicine, Seoul 05029, Republic of Korea; 5 Department of Medical Education, Konkuk University School of Medicine, Seoul 05029, Republic of Korea

**Keywords:** microglia, microbiota, neuropathic pain, allodynia, brain–gut axis, pregabalin

## Abstract

This study aimed to investigate the relationship between microglial activity and gut microbiota composition in a rat model of neuropathic pain (NP), and to evaluate how pregabalin treatment may influence these interrelated parameters. NP was simulated in rats via ligation and transection of the sciatic nerve. After confirming NP, the rats were randomly divided into treatment and control groups. Pregabalin (10 mg/kg) and the same dose of normal saline were administered to the treatment and control groups, respectively, on scheduled days. Microglial activity, cytokine levels, and the composition of the gut microbiota (assessed by the Firmicutes/Bacteroidetes (F/B) ratio) were evaluated. Pregabalin treatment significantly reduced microglial activity (which was notably lower in the treatment group than in the control group) and modulated pro-inflammatory and anti-inflammatory cytokine levels. While the F/B ratio in the control group significantly increased after NP surgery, the treatment group showed an initial increase followed by a notable decrease, approaching pre-surgery levels by day 28. This finding suggests that pregabalin treatment in rats with NP ameliorates microglial activity and is associated with a beneficial shift in the gut microbiota composition.

## 1. Introduction

The International Association for the Study of Pain defines neuropathic pain (NP) as “pain initiated or caused by a primary lesion or dysfunction in the nervous system” [1]. NP is characterized by allodynia and hyperalgesia and significantly impairs quality of life in affected individuals [2,3]. Although various therapeutic strategies have been introduced [4,5], the complex pathophysiology of NP continues to challenge effective treatment.

Microglia, the resident immune cells of the central nervous system (CNS), play a pivotal role in the development and maintenance of NP [6,7]. Upon peripheral nerve injury, microglia undergo phenotypic activation, contributing to central sensitization [8]. This “microglial activity” is typically evaluated using immunohistochemical markers such as ionized calcium-binding adapter molecule 1 (Iba1) and transmembrane protein 119 (TMEM119), which reflect morphological or phenotypic changes [9,10,11,12,13]. However, these markers do not fully capture the functional dimensions of microglial activation, such as cytokine release, phagocytosis, or modulation of synaptic transmission.

To better characterize the microglial activation state, functional readouts such as cytokine expression may serve as valuable complementary indicators. Cytokines are essential mediators of neuroimmune interactions. Pro-inflammatory cytokines such as interleukin-1β (IL-1β) and tumor necrosis factor-α (TNF-α) promote the M1 phenotype and facilitate pain hypersensitivity and central sensitization. In contrast, anti-inflammatory cytokines like IL-10 are associated with the M2 phenotype, supporting tissue repair and suppression of neuroinflammation. Therefore, cytokine profiling provides an additional layer of insight into microglial dynamics.

Increasing evidence also suggests that the gut microbiota influences neuroimmune responses through the gut–brain–immune axis. Dysbiosis in the gut microbiota has been linked to various CNS disorders, including NP [14,15,16]. Notably, changes in gut microbial composition can affect cytokine expression and microglial activation, thereby modulating pain states. To analyze microbial communities, 16S ribosomal RNA (rRNA) gene sequencing is a widely used technique that enables taxonomic profiling based on conserved and hypervariable regions of bacterial 16S rRNA genes.

Despite growing interest in the gut–brain axis, few studies have addressed how microglial activation and gut microbiota interact in the context of NP. This study aimed to investigate the relationship between microglial activity, cytokine expression, and gut microbiota composition in a rat model of NP, and to evaluate how pregabalin treatment may influence these interrelated parameters.

## 2. Results

The primary outcome was the composition of the microbiota (F/B) in the gut 28 days after surgery for NP, and the secondary outcome was microglial activity. A pilot study was performed with three rats each from the pregabalin and control groups. The pilot study showed that the microbiota composition, F/B, 28 days after surgery for NP were 0.85 ± 0.16 and 1.24 ± 0.18 for the treatment and control groups, respectively, and microglial activities were 28.04 ± 2.76% and 49.25 ± 9.64% for the treatment and control groups, respectively. Sample sizes of 12 for the primary outcome and 8 for the secondary outcome were calculated as appropriate to achieve a power of 0.9 and an α value of 0.05.

Twelve rats were enrolled in this study and evenly allocated into two groups. Surgery for the NP was successfully performed in all rats without any complications, and pregabalin or normal saline was administered without any adverse events.

**Figure 1:**
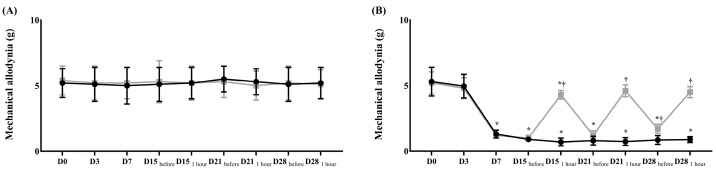
Assessment of mechanical allodynia for neuropathic pain before and after the treatment of normal saline or pregabalin. (**A**) Contralateral side for the surgery, (**B**) ipsilateral side for the surgery. (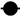) Control group, (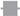) treatment group. Data are presented as means ± standard deviation (*n* = 6 per group). Statistical significance was determined by two-way repeated analysis of variance (ANOVA) followed by Tukey’s post hoc test for intra-group comparisons and unpaired *t*-test for inter-group comparisons. Abbreviations: Control, control group; treatment, treatment group; D0, before the surgery; D, day after the surgery; before, before the treatment of normal saline or pregabalin; after, 60 min after the treatment of normal saline or pregabalin. * *p* < 0.05 compared with D0 within the same group. † *p* < 0.05 compared with the control group at the same time point.

**Figure 2:**
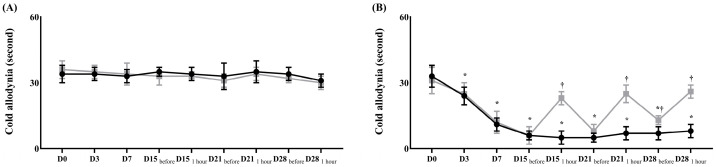
Assessment of cold allodynia for neuropathic pain before and after the treatment with normal saline or pregabalin. (**A**) Contralateral side for the surgery, (**B**) ipsilateral side for the surgery. (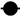) Control group, (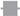) treatment group. Abbreviations: Control, control group; treatment, treatment group; D0, before the surgery; D, day after the surgery; before, before the treatment of normal saline or pregabalin; after, 60 min after the treatment of normal saline or pregabalin. Data are presented as means ± standard deviation (*n* = 6 per group). Statistical significance was determined by two-way repeated ANOVA followed by Tukey’s post hoc test for intra-group comparisons and unpaired t-test for inter-group comparisons.* *p* < 0.05 compared with D0 within the same group. † *p* < 0.05 compared with the control group at the same time point.

Before surgery, the contralateral and ipsilateral sides in both groups showed no differences in the withdrawal response to mechanical and cold allodynia (Figure 1 and Figure 2).

After surgery, the ipsilateral side in both groups showed significantly lower thresholds for mechanical stimulation and a significantly earlier withdrawal response to cold stimulation than the contralateral side (Figure 1 and Figure 2). After treatment with pregabalin or normal saline at 15, 18, 21, and 28 days after the surgery, the withdrawal response to a von Frey filament fiber and dry ice at the contralateral side did not significantly differ between the two groups (Figure 1 and Figure 2). However, both responses on the ipsilateral side were significantly less sensitive in the treatment group than in the control group (Figure 1 and Figure 2).

**Figure 3:**
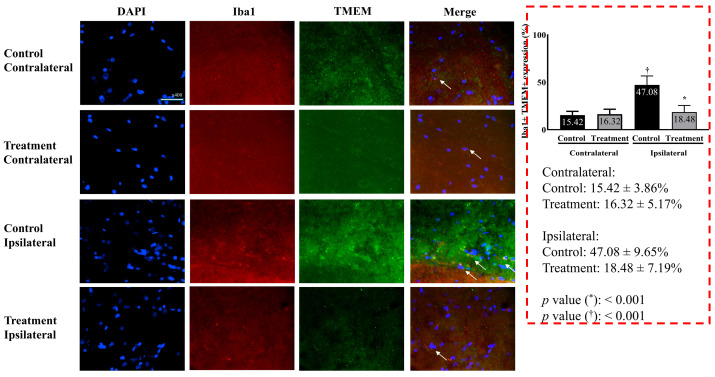
Microglial activity in the L5 segment of the spinal cord. Merged images represent overlay of Iba1 (green), TMEM119 (red), and DAPI (blue). Consistent with the quantitative data presented, microglial activation (indicated by Iba1 and TMEM119 expression) was markedly higher on the ipsilateral side in the control group compared to the attenuated activation observed in the treatment group. Images were taken under identical magnification and exposure settings using an upright microscope. Data are presented as means ± standard deviation (*n* = 6 per group). Statistical significance was determined by unpaired t-test with Bonferroni correction for inter-group comparisons (control vs. treatment for each side) and paired t-test for within-group comparisons (ipsilateral vs. contralateral within each group). Abbreviations: DAPI, 4,6-diamidino-2-phenylindole; Iba1, ionized calcium-binding adapter molecule 1; TMEM119, transmembrane protein 119; Merged, merged image of DAPI, Iba1, and TMEM119; control, control group; treatment, treatment group; contralateral, contralateral side for surgery; ipsilateral, ipsilateral side for the surgery. * *p* < 0.05 compared with the control group. † *p* < 0.05 compared with the contralateral side in each group.

Microglial activity differed significantly between the two groups. The treatment group showed significantly lower microglial activity than the control group (18.48 ± 7.19% vs. 47.08 ± 9.65% in the treatment and control groups, respectively, *p* < 0.001) (Figure 3).

Cytokine levels also significantly differed between the two groups. Pro-inflammatory cytokines showed significantly lower levels in the treatment group than the control group (TNF-α, 1002.00 ± 134.40 pg/mL in the treatment group vs. 1746.00 ± 139.30 pg/mL in the control group, *p* < 0.05; IL-1β, 118.20 ± 16.33 pg/mL in the treatment group vs. 385.40 ± 17.93 pg/mL in the control group, *p* < 0.05). On the contrary, anti-inflammatory cytokine levels were significantly higher in the treatment group than in the control group (IL-4, 10.33 ± 0.60 pg/mL in the treatment group vs. 2.80 ± 1.02 pg/mL in the control group, *p* < 0.05; IL-10, 280.10 ± 12.95 pg/mL in the treatment group vs. 168.50 ± 9.60 pg/mL in the control group, *p* < 0.05) (Table 1).

**Figure 4:**
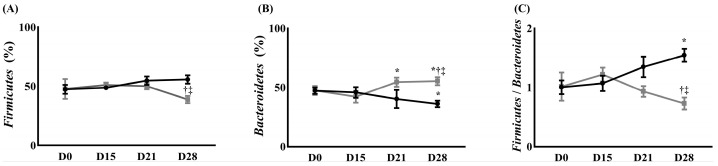
The change in gut microbiota composition. (**A**) Firmicutes, (**B**) Bacteroidetes, and (**C**) Firmicutes/Bacteroidetes ratio. (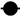) Control group, (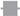) treatment group. Data are presented as means ± standard deviation (*n* = 6 per group). Statistical significance was determined by two-way repeated ANOVA (followed by Tukey’s post hoc test for within-group comparisons over time and for between-group comparisons at specific time points). Abbreviations: D0, before the surgery; D, day after the surgery. * *p* < 0.05 compared with D0 within the same group. † *p* < 0.05 compared with D15 within the same group. ‡ *p* < 0.05 compared with the control group at the same time point.

At the phylum level, the population of Firmicutes in the control group increased without any significance until 28 days after surgery for NP compared to before surgery for NP. The population of Firmicutes in the treatment group increased until 15 days after surgery for NP and then decreased compared with that before surgery for NP. It was significantly lower 28 days after the surgery, compared with that 15 days after the surgery (51.00 ± 2.00% on day 15 after surgery vs. 38.67 ± 1.15% on day 28 after surgery, *p* < 0.05). The result on day 28 significantly differed between the groups (38.67 ± 1.15% in the treatment group vs. 55.67 ± 3.51% in the control group, *p* < 0.05) (Figure 4A).

At the phylum level, the population of Bacteroidetes in the control group decreased until 28 days after surgery compared with that before the surgery. It was significantly lower 28 days after surgery for NP, compared with before surgery (47.3 ± 2.77% before surgery for NP vs. 36.13 ± 2.78% 28 days after surgery, *p* < 0.05). The population of Bacteroidetes in the treatment group decreased 15 days after surgery for NP and increased until 28 days after surgery for NP compared to before surgery for NP. The populations of Bacteroidetes in the treatment group were significantly higher 21 and 28 days after surgery for NP, compared with 15 days after the surgery [42.2 ± 5.00% 15 days after surgery for NP vs. 53.83 ± 5.06% and 55.33 ± 2.41% 21 and 28 days after surgery (*p* < 0.05), respectively]. The population of Bacteroidetes in the treatment group also significantly differed from the control group 28 days after the surgery (55.33 ± 2.41% in the treatment group vs. 36.13 ± 2.78% in the control group, *p* < 0.05) (Figure 4B).

The Firmicutes/Bacteroidetes (F/B) ratio in the control group increased until 28 days after surgery for NP compared with that before surgery (1.00 ± 0.12 before surgery vs. 1.55 ± 0.11 on 28 days after surgery for NP, *p* < 0.05). In contrast, the treatment group showed an increase in the F/B ratio 15 days after surgery for NP and decreased until 28 days after surgery for NP, compared to before surgery for NP. It was significantly lower 28 days after surgery compared with 15 days after surgery (1.22 ± 0.12 15 days after surgery vs. 0.78 ± 0.08 28 days after surgery, *p* < 0.05). This finding significantly differed from that of the control group (0.78 ± 0.08 in the treatment group vs. 1.55 ± 0.11 in the control group, *p* < 0.05) (Figure 4C).

## 3. Discussion

In this study, pregabalin, administered for the treatment of rats with NP, ameliorated mechanical and cold allodynia. Microglial activity was significantly suppressed after pregabalin administration to rats with NP. This treatment also led to significant changes in the gut microbiota population, including a normalized F/B ratio.

In NP, microglia activation induces their differentiation, resulting in increased pro-inflammatory (M1 phenotype) and decreased anti-inflammatory (M2 phenotype) microglia. It is associated with increased levels of pro-inflammatory cytokines such as TNF-α and IL-1β and decreased levels of anti-inflammatory cytokines such as IL-4 and IL-10. The alteration of cytokines is a known mechanism of nociceptive dysfunction in NP. Furthermore, microglial activity induces pain hypersensitivity [17]. In this study, we observed alterations in cytokine levels induced by microglial activity.

Previous studies also showed that pregabalin administration changed microglial differentiation in NP [18], although only microglial activity without microglial differentiation was checked in the present study. Pregabalin administration inhibited M1 phenotype expression and augmented M2 phenotype, and finally, improved mechanical and cold allodynia in rats with NP [18]. This suggests that increased microglial activities from the rats with NP in the present study were pro-inflammatory, considering cytokine release, and pregabalin administration inhibited them.

Recently, the finding that gut microbiota play a key role in homeostasis and that dysbiosis in the gut leads to various diseases has triggered further research to elucidate the mechanism of various diseases via the gut microbiota [19,20,21]. Several studies have shown that changes in the population of the gut microbiota occur after the occurrence of NP [14,15,16]. Improvement, rather than a simple change in the population of the gut microbiota, might be more clinically meaningful. Therefore, in the present study, we evaluated two groups based on the presence or absence of pregabalin for the treatment of NP.

The present study used pregabalin for NP treatment. Pregabalin regulates voltage-gated calcium channels of the nervous system and pain-related neurotransmitters. It is a first-line pharmacological treatment for patients with NP. Particularly, pregabalin stimulates the expression of IL-10 and β-endorphin in spinal microglia. This correlates with an improvement in mechanical allodynia [22]. In this study, we observed that pregabalin increases IL-10 levels and improves mechanical allodynia. We also found that pregabalin suppressed the expression of pro-inflammatory cytokines such as TNF-α and IL-1β. This indicated that the effect of pregabalin on microglial activity was associated with the stimulation of anti-inflammatory cytokines and the suppression of pro-inflammatory cytokines.

We focused on the F/B ratio to evaluate changes in the population of the gut microbiota, although several indices have been used [23]. Firmicutes represent gram-positive bacteria, while Bacteroidetes represent gram-negative bacteria [24]. Firmicutes and Bacteroidetes are the two phyla with the largest proportions of gut microbiota [23]. Previous studies have demonstrated that a high F/B ratio is associated with pathological conditions such as inflammation, CNS diseases, and several pain syndromes such as fibromyalgia and osteoarthritis [14,19,23,25,26,27,28]. Therefore, we used the F/B ratio to evaluate changes in the gut microbiota population.

In the present study, the significantly lower F/B ratio observed after pregabalin administration suggested that pregabalin not only affected microglial activity but also caused shifts in the gut microbiota composition, potentially reversing dysbiosis.

The observed cytokine profiles are consistent with a shift from M1-dominant pro-inflammatory microglial activation in untreated rats to a more anti-inflammatory phenotype in pregabalin-treated animals. This transition was accompanied by partial normalization of the F/B ratio, supporting a potential link between central immune activity and peripheral microbial composition.

While these findings highlight meaningful compositional changes at the phylum level, it is important to acknowledge the limitations of this resolution. Genus-level profiling and microbial diversity metrics (e.g., Shannon index, UniFrac distances) could provide more detailed ecological insights. However, such analyses require deeper sequencing and larger sample sizes, which were beyond the scope of the current study. Future investigations should incorporate these approaches to more comprehensively explore taxonomic and ecological alterations.

In addition, although a naive (non-operated) control group was not included, the sham-operated group served as a reference for evaluating the effect of sciatic nerve injury while minimizing additional animal use. The sham animals received identical surgical exposure without nerve ligation, thereby allowing valid comparisons to isolate the effects of neuropathic pain.

Despite the strengths of our findings, several limitations should be noted. First, we demonstrated associations rather than mechanisms. Specifically, we did not directly evaluate how changes in spinal microglial activity correlate with gut microbiota alterations. Further studies are needed to elucidate these mechanistic pathways. Second, the relatively small sample size used in this study may limit the generalizability of our findings and their statistical power, particularly for detecting subtle effects. Future studies with larger cohorts would be beneficial to confirm these results. Third, although the trend in the F/B ratio was informative, a quantitative correlation between spinal microglial activity and the gut F/B ratio could not be assessed because microglial analysis was performed only post-mortem.

In conclusion, the change in microglial activity in the spinal cord is significantly associated with the changes in the gut microbiota population during the occurrence and treatment of NP in rats. These findings contribute to our understanding of the pathogenesis of NP and suggest potential avenues for developing appropriate treatment modalities.

## 4. Materials and Methods

### 4.1. Ethics

All experiments were performed in accordance with the National Institute of Health Guidelines for the Care and Use of Laboratory Animals. After obtaining approval from the Institutional Animal Care and Use Committee (IACUC) of Konkuk University (approval number: KU22021, 23 February 2022), all experiments were conducted at the Laboratory Animal Research Center in accordance with the IACUC guidelines.

### 4.2. Animals

Male Sprague-Dawley rats of 6−8 weeks of age were purchased from Orient Bio (Seongnam, Korea). Rats were housed in cages with free access to water and food. The room was maintained with a standard light and dark cycle every 12 h, with lights on at 7:00 and lights off at 19:00 at a room temperature of 25 °C [29]. The rats were acclimated to the experimental conditions for 7 days before surgery for NP [30]. All experiments were performed during the daytime. Before surgery for NP, all rats were assessed for NP and allodynia. Surgery for NP was performed after evaluation confirmed the absence of NP. At 3, 7, and 14 days after the surgery, NP expression was evaluated using the same techniques as before surgery. If enough expression was not obtained during the assessment of NP, the rats were excluded from the experiment.

After confirming NP expression, the rats were randomly divided into two groups: treatment and control groups. At 15, 21, and 28 days after surgery for NP, pregabalin or normal saline was administered intraperitoneally according to allocation. Pregabalin (10 mg/kg) was mixed with normal saline (0.5 mL) and was administered to the treatment group. Equal volumes of normal saline were injected into the control group. NP was assessed before and 60 min after the administration of pregabalin or normal saline.

### 4.3. Assessment of NP

NP was assessed using mechanical and cold allodynia with von Frey filaments and dry ice, respectively. This was performed by an observer blinded to the allocation.

Mechanical allodynia was measured using von Frey filament fibers with forces of 0.6, 1.0, 1.4, 2.0, 4.0, 6.0, 8.0, 11.0, and 15.0 g. The rats were placed in a transparent test cage with a wire mesh metal floor for 30 min before the test. The rigid tip of the von Frey filament was applied perpendicular to the skin of the lateral plantar area of the left hind paw until it bent. The fiber of the von Frey filament with a force of 2.0 g was started. If a response such as paw withdrawal or paw licking occurred, a weaker force was applied using the Dixon up–down method. If no response occurred, a stronger force was applied using the Dixon up–down method [31,32]. A maximum force of 15.0 g was assigned as the threshold value.

Cold allodynia was measured using dry ice at −80 °C. The rats were placed in a transparent cage with a glass floor for 30 min before testing for environmental adaptation. The dry ice was ground into a fine powder using a hammer. The dry ice powder was filled in a 10 mL syringe and compressed until it could not be compressed any further. Using a syringe plunger, a pellet of dry ice was pushed until it was past 20−30 mm from the tip of the syringe. Dry ice pellets were gently and firmly applied to the glass beneath the hind paws of the rats. The withdrawal response was then measured.

### 4.4. Surgery for NP

Surgery for NP was established on the basis of previous studies [18,30,33]. Anesthesia was induced with 5% isoflurane (JW Pharmaceutical, Gwacheon-si, Korea) using oxygen (300 mL/min) and nitrous oxide (700 mL/min). After inducing anesthesia, the rats were moved to the surgical platform, and anesthesia was assessed by pinching the hindfoot. With the rat in a supine position, the tongue was pulled out using forceps. After intubation with a 1.77-inch-long 16-gauge, 4.5 cm catheter (BD, NJ, USA), the correct intubation position was confirmed by checking for symmetric chest expansion. A ventilator (Harvard Apparatus, Holliston, MA, USA) was connected to the intubation catheter and set to (1) a fraction of inspired O_2_ (FiO_2_) of 0.5, (2) inspiratory flow rate of 170 mL/min, (3) tidal volume of 6 mL/kg, and (4) respiratory rate of 80 breaths/min. Anesthesia was maintained with isoflurane via an intubation catheter using a 4.0% vaporizer. After intubating and setting the ventilator, the rats were gently moved to the prone position. The depth of the intubation catheter was rechecked with the rat in a prone position. After confirming the proper position for intubation, the surgery was performed. The left leg of each rat was fastened to the platform with tape, and the hairs around the left thigh were shaved. After sterilization with 70% alcohol around the shaved thigh, the sciatic nerve, along with three branches of the common peroneal nerve, tibial nerve, and sural nerve, was exposed through a 2 cm incision at the posterior side along the heel. The two nerves, except the sural nerve, were ligated with silk 5.0 and transected 2 mm distal to the ligation. After transection, the muscle and skin were sutured, and the incision site was disinfected. For the control group, a sham operation was performed, involving identical surgical exposure and muscle incision without nerve ligation or transection. Following the procedure, the muscle and skin were sutured, and the incision site was disinfected, similar to the neuropathic pain group.

### 4.5. Spinal Cord Preparation

After the last assessment of NP, 60 min after the administration of pregabalin or normal saline, the rats were transferred into an anesthesia induction chamber. Isoflurane (5% volume for anesthesia induction and 3% volume for anesthesia maintenance) in 300 mL/min oxygen and 700 mL/min nitrous oxide was used. During anesthesia, the rats were dissected, and the right atrium was exposed. For the exsanguination of whole blood, 1× phosphate-buffered saline (PBS) was administered to the right atrium. The spinal cord was extracted to evaluate microglial activity and degree of inflammation using enzyme-linked immunosorbent assay (ELISA) for cytokines.

### 4.6. Microglial Activity in the Spinal Cord

Double immunofluorescence staining was performed. The tissues on the slides were immersed in 5% normal goat serum (Abcam, Cambridge, MA, USA) for 1 h at room temperature to block nonspecific binding. After blocking, the tissues were washed three times with 1× PBS for 5 min and stained with the first antibodies of rabbit TMEM119 (Novus Biologicals, Centennial, CO, USA, 1:200) and mouse Iba1 (Thermo Fisher Scientific, Waltham, MA, USA, 1:1000) in 1% normal goat serum for 1 h at room temperature. After staining, rabbit immunoglobulin G (Bioss Antibodies, Woburn, MA, USA, 1:200) and mouse immunoglobulin G (Thermo Fisher Scientific, Waltham, MA, USA, 1:500) were added for 1 h. After staining, the tissues were washed three times with 1× PBS. After washing, the tissues were incubated with goat Alexa Fluor 488 (Thermo Fisher Scientific, Waltham, MA, USA, 1:1000) and goat Alexa Fluor 594 (Thermo Fisher Scientific, Waltham, MA, USA, 1:2000) secondary antibodies in 1% normal goat serum for 1 h at room temperature. After staining, the tissues were washed three times with 1× PBS. After washing, the tissues were stained with 4,6-diamidino-2-phenylindole (DAPI) for nuclear staining in the dark for 5 min at room temperature. After staining, the tissues were washed with 1× PBS for 5 min, and an anti-fade medium was added to the tissues. The tissues were covered with cover glasses and sealed with nail polish. For quantitative analysis, images were acquired using an upright microscope. Image analysis was performed under identical conditions using the Image J version 1.54 program (Image J, USA).

### 4.7. Cytokines in the Spinal Cord

The spinal cord was homogenized with cold 1× PBS and centrifuged at 12,000× *g* for 15 min at 4 °C. The supernatants were collected into the Eppendorf tubes and used for ELISA assay to assess pro-inflammatory TNF-α and IL-1β, and the anti-inflammatory cytokines IL-4 and IL-10. Cytokine levels in spinal cord tissue were measured using commercial ELISA kits: IL-1β (R&D Systems, Minneapolis, MN, USA, Cat# RLB00), TNF-α (R&D Systems, Minneapolis, MN, USA, Cat# 510-RT), and IL-10 (R&D Systems, Minneapolis, MN, USA, Cat# R1000). All assays were conducted following the manufacturer’s protocols.

### 4.8. Gut Microbiota

Fecal samples were collected to assess the gut microbiome at the following time points: (1) 1 h before surgery for NP, (2) 15 days after surgery for NP and before intraperitoneal administration of pregabalin or normal saline, (3) 21 days after surgery for NP and before intraperitoneal administration of pregabalin or normal saline, and (4) 28 days after surgery for NP. The samples were transferred into a 1.5 mL Eppendorf tube. The tube containing the sample was immediately frozen and stored in a deep freezer at -80 °C. Fecal DNA was extracted using the QIAamp PowerFecal DNA Kit (Qiagen, Hilden, Germany) following the manufacturer’s instructions. The V3–V4 hypervariable regions of the bacterial 16S rRNA gene were amplified using universal primers (341F and 805R). Sequencing was conducted on the Illumina MiSeq platform (Illumina, San Diego, CA, USA) using 2 × 300 bp paired-end reads. Subsequent 16S rRNA gene sequencing and bioinformatic analysis were performed by Humanizing Genomic Macrogen Inc. (Seoul, Korea). Raw sequences were processed using QIIME2 (version 2021.2), including quality filtering, denoising with DADA2, and chimera removal. Taxonomic assignment was performed using a pre-trained classifier with the SILVA 138 reference database. We also evaluated the F/B ratio.

### 4.9. Statistics

Statistical analyses were performed using GraphPad Prism software (ver. 5.01; GraphPad Software, La Jolla, CA, USA). Depending on the specific experimental design and comparison, data were analyzed using either unpaired *t*-tests, paired *t*-tests, or two-way repeated measures analysis of variance (ANOVA). For analyses involving multiple comparisons, appropriate post hoc tests were applied. Specifically, two-way repeated measures ANOVA was followed by Tukey’s post hoc test. When multiple inter-group comparisons were performed using unpaired t-tests, Bonferroni correction was applied as indicated in the figure legends. A *p*-value < 0.05 was considered statistically significant. Data are presented as means ± standard deviation.

## Figures and Tables

**Table 1:** Cytokines in the spinal cord.

	Control Group	Treatment Group	*p*-Value
Pro-inflammatory cytokines			
TNF-α (pg/mL)	1746.00 ± 139.30	1002.00 ± 134.40	<0.05
IL-1β (pg/mL)	385.40 ± 17.93	118.20 ± 16.33	<0.05
Anti-inflammatory cytokines			
IL-4 (pg/mL)	2.80 ± 1.02	10.33 ± 0.60	<0.05
IL-10 (pg/mL)	168.50 ± 9.60	280.10 ± 12.95	<0.05

Data are expressed as mean ± standard deviation (*n* = 6 per group). *p*-values for comparisons between control and treatment groups for each cytokine were determined by unpaired t-test with Bonferroni correction. A *p*-value < 0.05 was considered statistically significant. Abbreviations: TNF-α, tumor necrosis factor-α; IL, interleukin.

## Data Availability

The datasets generated during and/or analyzed during the current study are available from the corresponding author upon reasonable request.

## Abbreviations

The following abbreviations are used in this manuscript:

ANOVA	Analysis of variance
CNS	Central nervous system
DAPI	4,6-diamidino-2-phenylindole
ELISA	Enzyme-linked immunosorbent assay
F/B	Firmicutes/Bacteroidetes
IACUC	Institutional animal care and use committee
Iba1	Ionized calcium binding adaptor molecule 1
IL	Interleukin
NP	Neuropathic pain
PBS	Phosphate buffered saline
rRNA	Ribosomal RNA
TMEM	Transmembrane protein
TNF-α	Tumor necrosis factor-alpha

## References

[1] Finnerup N.B., Haroutounian S., Kamerman P., Baron R., Bennett D.L.H., Bouhassira D., Cruccu G., Freeman R., Hansson P., Nurmikko T. (2016). Neuropathic pain: An updated grading system for research and clinical practice. Pain.

[2] Jensen T.S., Finnerup N.B. (2014). Allodynia and hyperalgesia in neuropathic pain: Clinical manifestations and mechanisms. Lancet Neurol..

[3] Torta R., Ieraci V., Zizzi F. (2017). A Review of the Emotional Aspects of Neuropathic Pain: From Comorbidity to Co-Pathogenesis. Pain Ther..

[4] O’Connor A.B., Dworkin R.H. (2009). Treatment of neuropathic pain: An overview of recent guidelines. Am. J. Med..

[5] Bouhassira D., Attal N. (2023). Personalized treatment of neuropathic pain: Where are we now?. Eur. J. Pain.

[6] Tsuda M., Masuda T., Kohno K. (2023). Microglial diversity in neuropathic pain. Trends Neurosci..

[7] Karavis M.Y., Siafaka I., Vadalouca A., Georgoudis G. (2023). Role of Microglia in Neuropathic Pain. Cureus.

[8] Atta A.A., Ibrahim W.W., Mohamed A.F., Abdelkader N.F. (2023). Microglia polarization in nociplastic pain: Mechanisms and perspectives. Inflammopharmacology.

[9] Poppell M., Hammel G., Ren Y. (2023). Immune Regulatory Functions of Macrophages and Microglia in Central Nervous System Diseases. Int. J. Mol. Sci..

[10] Mika J., Zychowska M., Popiolek-Barczyk K., Rojewska E., Przewlocka B. (2013). Importance of glial activation in neuropathic pain. Eur. J. Pharmacol..

[11] Mika J., Osikowicz M., Makuch W., Przewlocka B. (2007). Minocycline and pentoxifylline attenuate allodynia and hyperalgesia and potentiate the effects of morphine in rat and mouse models of neuropathic pain. Eur. J. Pharmacol..

[12] Tsuda M., Masuda T., Tozaki-Saitoh H., Inoue K. (2013). Microglial regulation of neuropathic pain. J. Pharmacol. Sci..

[13] Mika J., Popiolek-Barczyk K., Rojewska E., Makuch W., Starowicz K., Przewlocka B. (2014). Delta-opioid receptor analgesia is independent of microglial activation in a rat model of neuropathic pain. PLoS ONE.

[14] Guo R., Chen L.H., Xing C., Liu T. (2019). Pain regulation by gut microbiota: Molecular mechanisms and therapeutic potential. Br. J. Anaesth..

[15] Chen P., Wang C., Ren Y.N., Ye Z.J., Jiang C., Wu Z.B. (2021). Alterations in the gut microbiota and metabolite profiles in the context of neuropathic pain. Mol. Brain.

[16] Pane K., Boccella S., Guida F., Franzese M., Maione S., Salvatore M. (2022). Role of gut microbiota in neuropathy and neuropathic pain states: A systematic preclinical review. Neurobiol. Dis..

[17] Tsuda M., Shigemoto-Mogami Y., Koizumi S., Mizokoshi A., Kohsaka S., Salter M.W., Inoue K. (2003). P2X4 receptors induced in spinal microglia gate tactile allodynia after nerve injury. Nature.

[18] Hong S.-W., Piao L., Cho E.-H., Seo E.-H., Kim S.-H. (2024). The Effect of Pregabalin on Microglia Differentiation in Rat with Neuropathic pain: A Preliminary Study. Int. J. Med. Sci..

[19] Shreiner A.B., Kao J.Y., Young V.B. (2015). The gut microbiome in health and in disease. Curr. Opin. Gastroenterol..

[20] Cockburn D.W., Koropatkin N.M. (2016). Polysaccharide Degradation by the Intestinal Microbiota and Its Influence on Human Health and Disease. J. Mol. Biol..

[21] Jandhyala S.M., Talukdar R., Subramanyam C., Vuyyuru H., Sasikala M., Nageshwar Reddy D. (2015). Role of the normal gut microbiota. World J. Gastroenterol..

[22] Ahmad K.A., Shoaib R.M., Ahsan M.Z., Deng M.Y., Ma L., Apryani E., Li X.Y., Wang Y.X. (2021). Microglial IL-10 and beta-endorphin expression mediates gabapentinoids antineuropathic pain. Brain Behav. Immun..

[23] Rinninella E., Raoul P., Cintoni M., Franceschi F., Miggiano G.A.D., Gasbarrini A., Mele M.C. (2019). What is the Healthy Gut Microbiota Composition? A Changing Ecosystem across Age, Environment, Diet, and Diseases. Microorganisms.

[24] Jeffery I.B., O’Toole P.W., Ohman L., Claesson M.J., Deane J., Quigley E.M., Simren M. (2012). An irritable bowel syndrome subtype defined by species-specific alterations in faecal microbiota. Gut.

[25] Mitrea L., Nemes S.A., Szabo K., Teleky B.E., Vodnar D.C. (2022). Guts Imbalance Imbalances the Brain: A Review of Gut Microbiota Association With Neurological and Psychiatric Disorders. Front. Med..

[26] Sirisinha S. (2016). The potential impact of gut microbiota on your health:Current status and future challenges. Asian Pac. J. Allergy Immunol..

[27] O’ Mahony S.M., Dinan T.G., Cryan J.F. (2017). The gut microbiota as a key regulator of visceral pain. Pain.

[28] Hao X., Shang X., Liu J., Chi R., Zhang J., Xu T. (2021). The gut microbiota in osteoarthritis: Where do we stand and what can we do?. Arthritis Res. Ther..

[29] Yamauchi Y., Qin L.H., Nishihara M., Sawada K., Kato K., Inoue S. (2005). Vulnerability of synaptic plasticity in the complexin II knockout mouse to maternal deprivation stress. Brain Res..

[30] Seo E.H., Piao L., Cho E.H., Hong S.W., Kim S.H. (2023). The Effect of Ketamine on Endoplasmic Reticulum Stress in Rats with Neuropathic Pain. Int. J. Mol. Sci..

[31] Chaplan S.R., Bach F.W., Pogrel J.W., Chung J.M., Yaksh T.L. (1994). Quantitative assessment of tactile allodynia in the rat paw. J. Neurosci. Methods.

[32] Deuis J.R., Dvorakova L.S., Vetter I. (2017). Methods Used to Evaluate Pain Behaviors in Rodents. Front. Mol. Neurosci..

[33] Decosterd I., Woolf C.J. (2000). Spared nerve injury: An animal model of persistent peripheral neuropathic pain. Pain.

